# Tree Biomass Sensitivity to Ozone Exposure: Insights From a Decade of Free‐Air Experiments

**DOI:** 10.1111/gcb.70728

**Published:** 2026-01-29

**Authors:** Annesha Ghosh, Andrea Viviano, Elena Paoletti, Yasutomo Hoshika, Elena Marra, Jacopo Manzini, Cesare Garosi, Matheus Casarini Siqueira, Barbara B. Moura

**Affiliations:** ^1^ Department of Life Science School of Natural Sciences, Central University of Jharkhand Ranchi Jharkhand India; ^2^ Institute of Research on Terrestrial Ecosystems (IRET), National Research Council Sesto Fiorentino Italy; ^3^ Department of Agricultural, Food, Environmental and Forestry Science and Technology (DAGRI) University of Florence Firenze Italy; ^4^ National Biodiversity Future Center (NBFC) Palermo Italy; ^5^ Department of Forest Biomaterials and Technology (SLU) Swedish University of Agricultural Sciences Umeå Sweden; ^6^ Environmental Research Institute São Paulo Brazil

**Keywords:** biomass allocation indices, carbon allocation, environmental pollution, LIF, O_3_, POD_1_, relative biomass, roots

## Abstract

Tropospheric ozone (O_3_) is a pervasive stressor that impairs forest biomass and alters carbon allocation strategies. This study assessed biomass responses across 17 woody taxa under free‐air controlled exposure (FACE), integrating a decade of experiments conducted with an analogous exposure regime applied to deciduous and evergreen species. The analysis provided a comparative evaluation of existing flux‐based metrics. Statistical analyses revealed consistent reductions in relative total (RTB), aboveground (RTAB), and belowground (RTBB) biomass with increasing O_3_ uptake in terms of phytotoxic ozone dose (POD_1_ mmol m^−2^). Deciduous species reached the 4% biomass reduction threshold (CL_4_) at lower POD_1_ levels for RTBB (10.21), RTAB (13.16), and RTB (10.77) and displayed relatively small ${△}_{{\mathrm{POD}}_{1}}$ values for RTBB (2.75), RTAB (5.70), and RTB (3.31), where ${△}_{{\mathrm{POD}}_{1}}$ represents the increment in O_3_ uptake required to reach the CL_4_ threshold. In contrast, evergreen species showed higher CL_4_ for RTBB (11.48), RTAB (15.40), and RTB (13.86) and larger ${△}_{{\mathrm{POD}}_{1}}$ values for RTBB (8.40), RTAB (12.32), and RTB (10.78), reflecting a slower biomass decline. Contrasting relationships suggest that leaf habit‐specific patterns are associated with divergent carbon allocation strategies under O_3_ stress. In deciduous species, POD_1_ and Leaf Index Flux (LIF) were negatively correlated with shoot‐to‐root ratio (S/R), whereas in evergreen species, both indices were positively correlated with leaf area ratio (LAR) and S/R. In conclusion, flux‐based metrics provided a biologically robust framework for quantifying O_3_‐induced biomass losses, revealing higher sensitivity in deciduous species than in evergreens and highlighting the root as the most vulnerable compartment under O_3_ exposure. The findings should be interpreted considering the spatial and temporal constraints of a single‐site FACE experiment and the focus on O_3_ as a stand‐alone stressor without interaction effects. Future research should combine O_3_ uptake with multi‐stressor frameworks to better predict biomass and carbon responses in complex field conditions.

## Graphical abstract

Using a decade of free‐air controlled exposure (FACE) experiments across 17 woody taxa, we evaluated biomass responses to O_3_ uptake using flux‐based metrics. Increasing phytotoxic ozone dose (POD_1_) caused consistent declines in relative total (RTB), aboveground (RTAB), and belowground (RTBB) biomass. Deciduous species reached the 4% biomass reduction threshold (CL_4_) at lower POD_1_ values and exhibited smaller ${△}_{{\mathrm{POD}}_{1}}$, indicating higher sensitivity, particularly in root biomass. Evergreen species showed higher CL_4_ values and larger ${△}_{{\mathrm{POD}}_{1}}$, reflecting slower biomass decline. Carbon allocation responses diverged by leaf habit: POD_1_ and Leaf Index Flux (LIF) were negatively associated with shoot‐to‐root ratio in deciduous species, but positively related to leaf area ratio and shoot‐to‐root ratio in evergreens. Overall, flux‐based metrics provide a biologically robust framework for quantifying O_3_‐induced biomass loss, highlighting the greater vulnerability of deciduous species and roots under chronic ozone exposure.

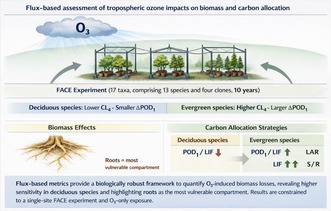

## 1 Introduction

In a period characterized by remarkable climate crises, the intensification of urbanization, land‐use changes, deforestation, and forest fires is linked with an increase in extreme atmospheric pollution events and a constant rise in atmospheric carbon dioxide (CO_2_) (Summerhayes and Zalasiewicz 2018). Among the most insidious pollutants, tropospheric ozone (O_3_) poses a significant threat, capable of penetrating biological tissues and inducing oxidative stress with adverse consequences for both terrestrial ecosystems and human health (Booker et al. 2009; Nuvolone et al. 2018).

The deleterious effects of O_3_ on plant health are related to visible foliar injury (VFI), photosynthetic impairment, reduced growth, and declining biomass yields (Emberson et al. 2018; Ghosh et al. 2020; Moura et al. 2022). A recent meta‐analysis of forest tree species studied in China, including both deciduous and evergreen taxa across temperate and subtropical regions, showed that elevated O_3_ concentrations (116 ppb) resulted in a 14% reduction in total tree biomass compared to control conditions (21 ppb) (Li et al. 2017). However, only a small fraction of the available evidence comes from free‐air experiments, and chamber‐based studies can introduce artefacts (Feng, Büker, et al. 2018; Hoshika, Agathokleous, et al. 2024). Therefore, the role of O_3_ in compromising plants' capacity to sequester carbon under real‐world conditions remains insufficiently explored. These knowledge gaps are particularly relevant under Mediterranean‐type climates, where high irradiance, recurrent summer drought, and elevated background O_3_ concentrations interact to shape species‐specific functional strategies and carbon allocation patterns in woody plants (Paoletti 2006).

Stomatal conductance is a crucial metric for assessing plant responses to O_3_ (Hoshika, Agathokleous, et al. 2024). When O_3_ enters leaves through the stomata, it induces the production of reactive oxygen species (ROS) that damage vital leaf functions, leading to declines in photosynthetic rate, chlorophyll content, and photochemical efficiency (Feng et al. 2020). The deterioration of the photosynthetic system reduces CO_2_ uptake, limiting carbon and energy availability for growth (Hoshika et al. 2022). These impairments not only threaten forest productivity but also disrupt the fragile balance of global carbon cycles, creating serious challenges for climate mitigation efforts (Cotrozzi 2021).

Traditional O_3_ risk assessments have mainly relied on concentration‐based metrics, such as AOT_40_, which quantify ambient O_3_ exposure without accounting for the actual dose absorbed by plants (Paoletti 2006). In contrast, physiological flux‐based metrics, including the phytotoxic ozone dose above a threshold y (PODy) and the leaf index flux (LIF), integrate stomatal O_3_ uptake with ecophysiological and morphological traits, providing a more mechanistic understanding of O_3_ stress dynamics (Sicard et al. 2016; Hoshika et al. 2017; Feng, Uddling, et al. 2018; Manzini et al. 2023; Manzini, Garosi, et al. 2025; Marra et al. 2025). Despite these advances, the relative effectiveness of these metrics in predicting biomass loss and alterations in carbon allocation patterns across woody species remains insufficiently explored.

Vegetation sensitivity to O_3_ occurs when pollutant concentrations exceed specific thresholds, potentially eliciting direct deleterious effects (Paoletti et al. 2020). In this context, critical levels (CLs) have been defined as “the atmospheric concentrations of pollutants above which adverse effects on receptors, such as humans, plants, ecosystems, or materials, may occur according to present knowledge” (UNECE 1996). For plants, CLs are currently based on stomatal flux‐derived indices (e.g., PODy) formulated within the Convention on Long‐Range Transboundary Air Pollution (CLRTAP) framework, with a potential effect at CL_4_ for woody species, corresponding to a 4% reduction in aboveground, root, or total tree biomass (Mills et al. 2017).

Evergreen and deciduous species exhibit distinct sensitivities and allocation strategies that reflect their contrasting ecophysiological adaptations (Carriero, Emiliani, et al. 2015; Feng, Uddling, et al. 2018). Among the functional traits, leaf area ratio (LAR) and shoot‐to‐root ratio (S/R) serve as integrative metrics of plant carbon economy, reflecting both the capacity for carbon assimilation and the efficiency of allocation across above‐ and belowground compartments. LAR largely determines the photosynthetic surface area available per unit of biomass and accounts for much of the inherent variation in relative growth rate (Poorter and van der Werf 1998), while recent evidence highlights its role within a whole‐plant coordination system that optimizes water–carbon trade‐offs (Gessler and Zweifel 2024). Likewise, S/R represents an important parameter for evaluating carbon distribution across plant compartments (Yang et al. 2018) and for interpreting phenotypic adjustments to environmental constraints (Freschet et al. 2015). Together, these traits offer an important framework to explore how O_3_ exposure may perturb the equilibrium between carbon gain and allocation, ultimately influencing plant growth and resilience.

These strategies are governed by a balance between below‐ and aboveground functions, where roots provide mechanical support, regulate water and mineral uptake, and store carbon in the long term, while aboveground parts primarily drive photosynthesis and canopy functions (Shang et al. 2017; Li et al. 2022). When this balance is disrupted, such as by O_3_ exposure, plants often prioritize shoot growth over root development, leading to changes in R/S and potentially undermining ecosystem stability (Shang et al. 2017; Li et al. 2022). In fact, belowground compartments are often more sensitive to O_3_ than aboveground tissues (Shang et al. 2017). At broader scales, a systematic reduction in root investment can constrain water and nutrient uptake, weaken plant–soil feedbacks, and reduce belowground carbon inputs, thereby increasing ecosystem vulnerability to additional stressors and potentially undermining ecosystem stability (Ping et al. 2023; Srivastava and Yetgin 2024).

Scientific evidence underscores the urgency of deepening our understanding of O_3_ effects on forest ecosystems, not only to preserve their ecological functionality but also to optimize climate change mitigation strategies (Ainsworth et al. 2012; Anav et al. 2016; Li et al. 2017).

This study evaluated the predictive power of O_3_ flux‐based metrics (POD_1_ and LIF) compared with the traditional concentration‐based index (AOT_40_) in explaining biomass reduction across 17 taxa, comprising 13 species and four clones (two clones of *Cupressus sempervirens* and two hybrid poplar clones; see Section 2.3 for details). The analysis was based on data from a decade‐long free‐air controlled exposure (FACE) series of experiments under realistic O_3_ conditions. In addition to assessing total biomass loss, we investigated how O_3_ exposure affects carbon allocation among roots, stems, and leaves, highlighting ecophysiological trade‐offs underlying resilience differences between evergreen and deciduous species as defined by leaf habit‐specific patterns. Furthermore, we determined critical O_3_ exposure thresholds, measured as CL_4_, varying by compartment (total, belowground, and aboveground biomass), predictor (POD_1_ and LIF), and leaf type (evergreen and deciduous), to better understand species sensitivity to O_3_ stress.

We hypothesized that flux‐based metrics (e.g., POD_1_ and LIF) would better capture biologically meaningful O_3_ effects on biomass than concentration‐based indices. We also expected that evergreen and deciduous species would show distinct patterns in O_3_ sensitivity and carbon allocation, driven by differences in key mechanistic ecophysiological traits related to leaf structure (e.g., leaf mass per area, LMA) and biomass allocation, as captured by biomass‐derived indices such as LAR and shoot‐to‐root ratio (S/R). Finally, we predicted that O_3_ stress would induce marked shifts in carbon allocation between belowground and aboveground compartments. To test these hypotheses, the study had four main aims: (i) evaluate the interrelationships among O_3_ metrics (POD_1_, LIF, AOT40) and biomass reductions (i.e., relative total biomass [RTB], relative total aboveground biomass [RTAB], and relative total belowground biomass [RTBB]) to identify the most physiologically meaningful predictor for subsequent modelling and CL_4_ estimation; (ii) identify leaf habit‐specific biomass response patterns to O_3_ exposure, focusing on differences between evergreen and deciduous trees; (iii) determine the critical levels for a 4% biomass reduction (CL_4_) across compartments, predictors, and leaf types to assess O_3_ sensitivity thresholds; (iv) investigate the ecophysiological mechanisms underlying O_3_‐induced alterations in biomass allocation patterns in shoot‐to‐root ratios and LAR.

## 2 Materials and Methods

### 2.1 Experimental Infrastructure

The O_3_ exposure experiments were conducted at FO_3_X (Free‐air O_3_ eXposure), a facility located in Sesto Fiorentino, central Italy, designed to investigate the effects of tropospheric O_3_ on vegetation under semi‐controlled environmental conditions. The facility consists of nine experimental plots (three plots per treatment), which simulate O_3_ regimes in which O_3_ is delivered via laser‐perforated micro‐holes for continuous exposure based on atmospheric levels (Paoletti et al. 2017). During the 2015 experiments, plants were exposed to ambient O_3_ concentrations (AA), as well as to 1.2 × AA and 1.4 × AA. Starting from 2016, the exposure levels were adjusted to include AA, 1.5 × AA, and 2.0 × AA.

### 2.2 Environmental Data Collection

At the FO_3_X facility, meteorological parameters are continuously recorded on an hourly basis (WatchDog Model 2000, Spectrum Technologies, Aurora, Illinois). Soil water content is quantified using EC5 probes connected to EM5b logging devices (Decagon Devices, Pullman, Washington). Atmospheric O_3_ concentrations are continuously monitored at vegetation height (approximately 1 m) using Model 202 analysers (2B Technologies, Boulder, Colorado). O_3_ is recorded at 10‐s intervals to produce hourly mean values. Calibration procedures are performed on all monitoring equipment before the start of each experimental campaign.

### 2.3 Plant Material

The target woody plants exposed to O_3_ included both evergreen and deciduous species, capable of living in the Mediterranean climate. A total of 281 single observations of evergreen species were considered. These comprised *Arbutus unedo* L. (*n* = 27), *Phillyrea angustifolia* L. (*n* = 27), *Quercus ilex* L. (*n* = 27), *C. sempervirens* L. clones 2546 (*n* = 38) and 3375 (*n* = 38), *Pinus pinea* L. (*n* = 44), *Pinus halepensis* Mill. (*n* = 45), and *Schinus terebinthifolia* Raddi (*n* = 35), a tropical species with notable capacity to adapt to Mediterranean environments (Nilsen and Muller 1980). In addition, a total of 468 single observations of deciduous species were examined. These included *Carpinus betulus* L. (*n* = 27), *Ostrya carpinifolia* Scop. (*n* = 27), the Oxford poplar clone ( *Populus maximowiczii* × *P. berolinensis*) (*n* = 270), poplar clone I‐214 ( *P. deltoides* W. Bartram ex Marshall × *P. nigra L*.) (*n* = 38), *Quercus pubescens* Willd. (*n* = 27), *Quercus robur* L. (*n* = 27), *Robinia pseudoacacia* L. (*n* = 21), and *Vaccinium myrtillus* L. (*n* = 31). Further details are provided in Table S1.

The exposure campaigns lasted for one growing season. At the end of each O_3_ exposure campaign, plants were harvested and separated into leaves, stems, and roots. Each part was oven‐dried at 80°C until the weight stabilized. The dry mass was measured using an analytical balance. Biomass values were summed to get the total above (stem and leaves) and below (root) ground biomass for each plant. All potted plants included in this study were irrigated daily to field capacity to prevent water stress.

### 2.4 Ozone Indices and Leaf Mass Per Area Measurement

Three exposure metrics were considered: AOT40, POD_1_, and LIF. AOT40 was calculated as the cumulative sum of hourly mean O_3_ concentrations exceeding 40 ppb, recorded during daylight hours when shortwave solar radiation exceeded 50 W m^−2^, over the course of each exposure period. In parallel, stomatal O_3_ flux was estimated on an hourly basis throughout the entire exposure period. A threshold of 1 nmol O_3_ m^−2^ s^−1^ was applied for calculating the cumulative phytotoxic O_3_ dose (POD_1_) as recommended by Mills et al. (2017). Both AOT40 and POD_1_ were calculated in accordance with the CLRTAP (Mills et al. 2017) protocols and are in line with the procedures adopted in previous studies (Finco et al. 2017; Hoshika et al. 2020; Moura et al. 2023; Viviano et al. 2025).

The LIF was calculated for each species and clone as the ratio between its mean POD_1_ and corresponding LMA. Originally, this index was applied to predict VFI and ecophysiological responses (Manzini, Garosi, et al. 2025; Manzini, Hoshika, et al. 2025); here, we investigated its correlation with Biomass Allocation Indices.

LMA values (g m^−2^) for all species, except for the *C. sempervirens* clones and *S. terebinthifolia*, were obtained from previous FO_3_X research papers. Newly generated LMA data for *C. sempervirens* clones 2546 and 3375 and for *S. terebinthifolia* were obtained following the same methodology described by Scartazza et al. (2025). For the broadleaf species *S. terebinthifolia*, five leaf discs of known area (0.50 cm^2^; 0.8 cm diameter) were taken from each sampled leaf with a leaf punch (Fujiwara Scientific Company Co. Ltd., Tokyo, Japan). Three leaves were collected per tree across nine trees. For the conifer *C. sempervirens* , three sun‐exposed twigs were gathered from each of three trees. The projected twig area was measured using the easy leaf area application (Easlon and Bloom 2014). The leaf discs and twigs were dried in an oven at 70°C until they reached a constant weight. Species‐specific LMA was then calculated as the ratio of the mean dry mass of leaf discs or twigs to their corresponding area. Mean O_3_ exposure indices are summarized in Table S2a, while LMA values and all corresponding literature references are provided in Table S2b.

### 2.5 Relative Biomass and Allocation Indices

Relative total biomass (RTB), RTAB, and RTBB were calculated as the ratio of the observed biomass to the reference biomass (*B*_ref_), following the approach described by Paoletti et al. (2017). In detail, *B*_ref_ was derived from the linear regressions between 24‐h averaged O_3_ concentration (M24, ppb) and each biomass component (Table S3), where *B*_ref_ represents the predicted biomass at a pre‐industrial O_3_ level of 20 ppb, as proposed by Tarasick et al. (2019). Biomass allocation indices such as LAR (total leaf area per total plant dry biomass) and shoot‐to‐root ratio (S/R, shoot mass [sum of leaves + stem dry mass] vs. root dry mass) were calculated as described by Poorter et al. (2012).

### 2.6 Statistical Analyses

#### 2.6.1 Premodeling Assessment

Pairwise associations among the physiological (i.e., RTB, RTAB, RTBB) and exposure variables (POD_1_, AOT40, LIF) were first evaluated using Spearman's rank correlation coefficients (*ρ*) to account for potential non‐linear relationships. Significance was assessed at *p <* 0.05, and 95% confidence intervals were estimated using 1000 bootstrap replicates.

To evaluate the suitability of the dataset for principal component analysis (PCA), the Kaiser–Meyer–Olkin (KMO) measure of sampling adequacy and Bartlett's test of sphericity were applied. Variables with individual KMO values < 0.5 were considered unsuitable for PCA and excluded from further multivariate analysis.

The PCA was then performed on the standardized variables (*z*‐scores), without rotation, to explore the dimensional structure among the retained predictors. Components with eigenvalues > 1 were extracted according to the Kaiser criterion, and loadings > |0.5| were considered relevant for component interpretation.

#### 2.6.2 Generalized Estimating Equations Models

We used generalized estimating equations (GEE) to analyze the relationship between relative biomass (i.e., RTB, RTAB, RTBB) and cumulative O_3_ uptake (POD_1_). Given the positive and right‐skewed distribution of the response variables, models were fitted with a Gamma family and log link, assuming an exchangeable correlation structure and clustering by species with robust standard errors. Model performance was assessed through residual diagnostics and a leave‐one‐species‐out (LOSO) sensitivity analysis to evaluate species influence on the estimated POD_1_ effect.

To assess differences in O_3_ sensitivity between evergreen and deciduous trees, analyses were stratified by leaf habit (Lh). We fitted the same GEE structure (Gamma family, log link), including an interaction term between POD_1_ and leaf habit (POD_1_ × Lh). The binary indicator Lh was coded as 0 = Deciduous (reference) and 1 = Evergreen, so the main effect of POD_1_ refers to deciduous species and the interaction term represents the change in slope for evergreens. Ninety‐five percent confidence intervals were derived from the fitted covariance matrix, and CL_4_ was calculated as the POD_1_ value associated with a 4% reduction from the maximum predicted response following the CLRTAP manual (Mills et al. 2017). The response sensitivity (Δ) was quantified as the change in POD_1_ needed to lower the response from its estimated maximum to the critical level CL_4_, defined as the difference between the POD_1_ at CL_4_ and the POD_1_ at the peak of the fitted model curve.

Model reliability was evaluated through convergence diagnostics, inspection of the working correlation structure, and formal heteroscedasticity tests (Breusch–Pagan). All analyses were performed in Python using the *statsmodels* package.

#### 2.6.3 Spearman Rank Correlation Analysis of Biomass Allocation Traits

Spearman rank correlation (*ρ*) coefficients were calculated to assess relationships between O_3_ exposure indices (POD_1_ and LIF) and plant biomass allocation traits (LAR and S/R). Analyses were conducted for the entire dataset and separately for deciduous and evergreen species groups. Statistical significance was evaluated at *α* = 0.05, with thresholds denoted as *p < 0.05, **p < 0.01, and ***p < 0.001. Only correlations with *p <* 0.05 were retained. Correlation patterns were visualized using Sankey diagrams, with link width proportional to correlation strength and color indicating direction (positive/negative) and O_3_ index. All analyses were performed in Python using *scipy.stats* and *scipy.spatial.distance* package.

## 3 Results

### 3.1 Cypress O_3_ Indices and Leaf Mass Per Area

We show here the results of cypress as they were unpublished. LMA values were similar between the two *C. sempervirens* clones. Clones 2546 and 3375 exhibited an average LMA of 424.82 and 434.08 g/m^2^, respectively (Table S2b).

Ozone exposure indices varied across treatments but remained generally similar between the two clones (Table S2b). For clone 2546, POD_1_ values were 10.39 mmol m^−2^ in AA, 15.82 mmol m^−2^ under 1.5 × AA, and 19.96 mmol m^−2^ in 2.0 × AA conditions. Corresponding LIF (mmol/g) values were 0.0222, 0.0440, and 0.0470, while AOT40 increased from 48.70 to 176.39 ppm·h across the same treatments. Clone 3375 exhibited identical POD_1_ and AOT40 values across treatments (10.39, 15.82, and 19.96 mmol m^−2^ for POD_1_; 48.70, 112.98, and 176.39 ppm h for AOT40), while its LIF values showed greater variation, rising from 0.0230 (AA) to 0.0386 (1.5 × AA) and peaking at 0.2953 under 2.0 × AA.

### 3.2 RTB, RTAB, and RTBB Pooled Model Analysis

Spearman's correlations and principal component analysis (PCA) were first used to identify the O_3_ metric most closely associated with biomass responses. Both POD_1_ and LIF showed moderate to strong negative correlations with RTB, RTAB, and RTBB (*ρ* = −0.25 to −0.48, *p <* 0.001), while AOT40 displayed only weak negative associations (*ρ* ≈−0.16 to −0.21, *p <* 0.001). The Kaiser–Meyer–Olkin test indicated a marginal overall sampling adequacy (KMO = 0.509), with a particularly low individual Measure of Sampling Adequacy for AOT40 (MSA = 0.178). Bartlett's test of sphericity was significant (*χ* ^2^ = 5552.6, df = 15, *p <* 0.001), supporting the suitability of the correlation matrix for factor extraction. PCA extracted two components with eigenvalues > 1, explaining 78.8% of the total variance. PC1 (56.5%) represented a physiological–O_3_ response axis, with high positive loadings for RTB (0.917), RTBB (0.891), and RTAB (0.799), and strong negative loadings for POD_1_ (−0.743) and LIF (−0.699). PC2 (22.3%) captured shared variability between POD_1_ (0.644) and LIF (0.693). The high uniqueness of AOT40 (0.916) confirmed its limited contribution to the multivariate structure.

**FIGURE 1:**
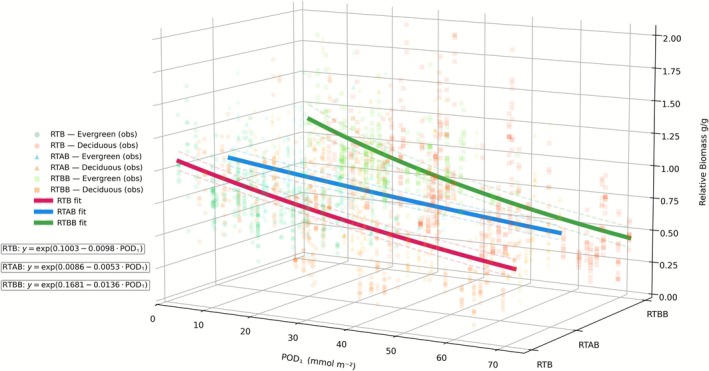
Relationship between phytotoxic ozone dose (POD_1_) and relative biomass (relative total biomass [RTB], relative total aboveground biomass [RTAB], and relative total belowground biomass [RTBB]) across 749 observations. Data points are shaped according to relative biomass (circular for RTB, triangle for RTAB, square for RTBB) and color‐coded by leaf type (green for evergreen or orange for deciduous). Solid lines represent fitted values from Gamma–log GEE models clustered by woody species, while dashed lines indicate 95% confidence intervals.

Consistent with these results, GEE models were applied using POD_1_ as the sole, physiologically relevant O_3_ predictor to assess its effects on biomass components. The models (Figure 1; Table S4) revealed a consistent and statistically significant negative association between POD_1_ and all three endpoints (RTB, RTAB, RTBB), indicating a general decline in relative biomass with increasing O_3_ uptake. This pattern was also reflected in the non‐parametric Spearman correlations, which yielded negative coefficients for all endpoints: RTB (*ρ* = −0.44), RTAB (*ρ* = −0.30), and RTBB (*ρ* = −0.48), based on 749 observations. These values confirm the expected inverse relationship, whereby higher POD_1_ exposure corresponded to lower biomass.

For RTB, the model yielded a significant intercept (0.1003; SE = 0.036; *z* = 2.77; *p* = 0.006) and a strongly negative coefficient for POD_1_ (−0.0098; SE = 0.001; *z* = −9.93; *p <* 0.001), confirming a consistent downward trend. In the case of RTAB, the intercept was not statistically significant (0.0086; SE = 0.035; *p* = 0.807), yet the effect of POD_1_ remained highly significant (−0.0054; SE < 0.001; *z* = −11.72; *p* < 0.001), although with a smaller magnitude. RTBB exhibited the largest effect size for POD_1_ (−0.0136; SE = 0.002; *z* = −8.33; *p* < 0.001), suggesting that belowground biomass was the most sensitive to O_3_ exposure, with the steepest predicted decline.

Residual diagnostics, including distributional plots and residuals versus fitted values (Figure S1), showed no systematic deviations from model assumptions, confirming the adequacy of the Gamma–log specification. Residuals displayed asymmetric but coherent distributions, and their dispersion around fitted values appeared random and homoscedastic, indicating that the model structure appropriately captured the underlying data.

### 3.3 Ozone Effects on RTB, RTAB, and RTBB in Evergreen and Deciduous Species

**FIGURE 2:**
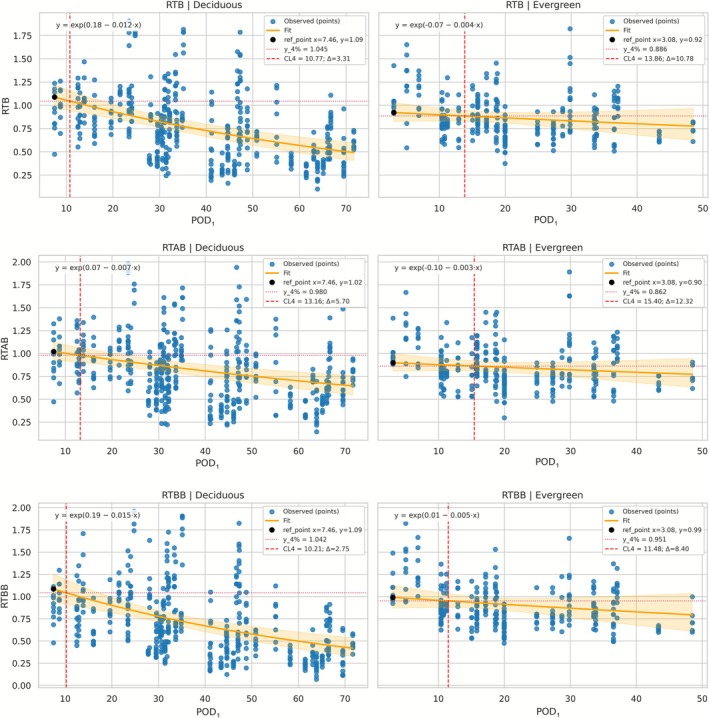
Generalized estimating equation (GEE) models describing the relationships between the phytotoxic ozone dose (POD_1_) and relative biomass for total (RTB), aboveground (RTAB), and belowground (RTBB) compartments across deciduous and evergreen species. Each panel presents modelled responses obtained from Gamma‐family GEE fits with a log link (orange line), and 95% confidence intervals (shaded area). Vertical dashed red lines mark the critical level for a 4% reduction in biomass (CL_4_–POD_1_ in mmol m^−2^), while black dots indicate the maximum predicted biomass (ref_point). The fitted exponential regression equations are shown within each panel.

The GEE models (Gamma distribution, log link) revealed consistent yet distinct patterns of O_3_ sensitivity across biomass components (Figure 2). For RTB, a significant main effect of POD_1_ (*p* < 0.001) and a significant interaction with leaf habit (POD_1_ × Lh: *β* = +0.009 ± 0.004, *p* = 0.013) indicated divergent responses between deciduous and evergreen species. Deciduous species exhibited a marked decline in RTB with increasing POD_1_ (*β* = −0.0124 ± 0.0021, *p* < 0.001), with a CL_4_–POD_1_ of 10.77 mmol m^−2^ (Δ = 3.31 POD_1_ units), whereas evergreens showed a weaker decline (effective *β* = −0.0037, *p* < 0.05) and a lower baseline biomass (Lh: *β* = −0.246 ± 0.091, *p* = 0.007), with a CL_4_–POD_1_ of 13.86 mmol m^−2^ (Δ = 10.78). For RTAB, POD_1_ had a significant negative effect (*β* = −0.0072 ± 0.0017, *p* < 0.001) without significant interaction with leaf habit (POD_1_ × Lh: *β* = +0.0038 ± 0.0032, *p* = 0.229), indicating a broadly consistent reduction across species. Deciduous trees showed a pronounced decrease (CL_4_–POD_1_ = 13.16 mmol m^−2^, Δ = 5.70), while evergreens exhibited a milder response (effective *β* ≈−0.0034; Lh: *β* = −0.171 ± 0.083, *p* = 0.038; CL_4_–POD_1_ = 15.40 mmol m^−2^, Δ = 12.32). For RTBB, the model identified a significant main effect of POD_1_ (*β* = −0.0148 ± 0.0028, *p* < 0.001) and a significant interaction with leaf habit (POD_1_ × Lh: *β* = +0.0100 ± 0.0041, *p* = 0.014). Deciduous species showed a steep decline in RTBB (*β* = −0.0148 ± 0.0028, *p* < 0.001; CL_4_–POD_1_ = 10.21 mmol m^−2^, Δ = 2.75), whereas evergreens displayed a more attenuated decrease (effective *β* ≈−0.0048; Lh: *β* = −0.187 ± 0.119, *p* = 0.114; CL_4_–POD_1_ = 11.48 mmol m^−2^, Δ = 8.40).

Model diagnostics across all GEE analyses confirmed strong convergence, homoscedastic residual patterns (B‐P for RTB = 0.4; RTAB = 0.6; RTBB = 0.2), low dispersion values (0.13; 0.12–0.19 respectively), and minimal intra‐cluster correlations (*ρ* ≈0.04; 0.08; 0.02 respectively), supporting the robustness and reliability of the parameter estimates.

Leave‐one‐species‐out (LOSO) analyses confirmed the robustness of CL_4_ estimates within the deciduous group, while revealing pronounced and structurally consistent interspecific heterogeneity among evergreen species. Across all biomass components, deciduous species showed relatively stable CL_4_–POD_1_ estimates, with limited dispersion across LOSO iterations (RTB: mean = 11.00 mmol m^−2^, SD = 0.55; RTAB: mean = 13.08 mmol m^−2^, SD = 1.24; RTBB: mean = 10.73 mmol m^−2^, SD = 0.66), indicating that group‐level responses were not driven by any single taxon. Exclusion of the O_3_‐sensitive Oxford poplar clone resulted in higher, though modest, deciduous CL_4_–POD_1_ estimates across all biomass compartments, increasing from 10.77 to 12.22 mmol m^−2^ for RTB, from 13.16 to 15.73 mmol m^−2^ for RTAB, and from 10.21 to 12.22 mmol m^−2^ for RTBB. Overall variability remained modest and Δ values clustered tightly (RTB: mean = 3.38; RTAB: 5.47; RTBB: 3.11), supporting a coherent deciduous sensitivity pattern.

In contrast, evergreen species exhibited pronounced interspecific heterogeneity, with LOSO‐derived CL_4_–POD_1_ values spanning wide ranges (RTB: 8.24–40.27 mmol m^−2^; RTAB: 9.11–40.24 mmol m^−2^; RTBB: 7.17–40.21 mmol m^−2^) and large standard deviations (SD ≈10–11 mmol m^−2^). This variability was consistently driven by *A. unedo* , whose exclusion resulted in marked upward shifts of group‐level CL_4_ estimates. Correspondingly, Δ values displayed wider dispersion among evergreen species (RTB: mean = 5.33; RTAB: 6.54; RTBB: 3.58) compared with deciduous species. Despite this heterogeneity, the relative contrast in O_3_ sensitivity (observing lower CL_4_–POD_1_ and Δ values in deciduous than in evergreen) was preserved across all LOSO iterations and biomass compartments.

### 3.4 Correlation Between Biomass Indices and O_3_

**FIGURE 3:**
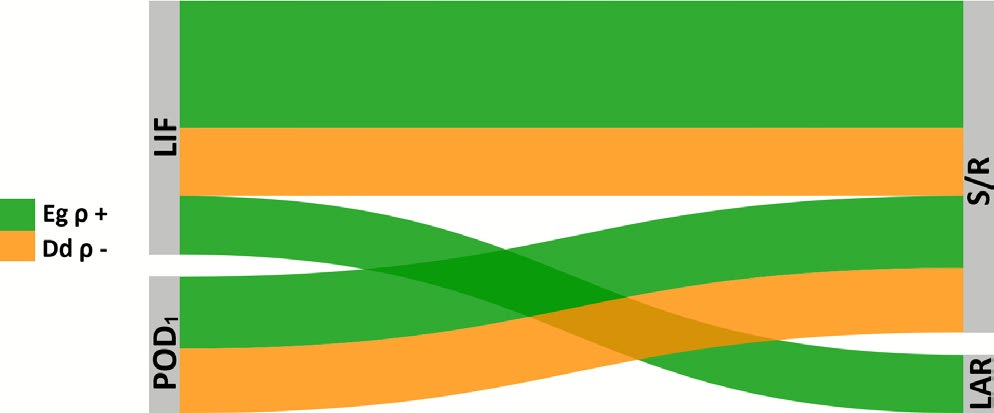
Sankey diagram illustrating Spearman correlations between ozone exposure and biomass allocation indices for deciduous species (Dd), and evergreen species (Eg). Variables include O_3_ stress indices (POD_1_, LIF) and the biomass allocation traits leaf area ratio (LAR), and shoot‐to‐root ratio (S/R). Links represent statistically significant correlations (only for *p* < 0.05), with line width proportional to the distance correlation values, and line color indicates the direction of the correlation (green only for positive *ρ* in Eg, orange for negative *ρ* in Dd).

The Spearman correlation analysis revealed significant associations between O_3_ exposure indices (POD_1_, LIF) with LAR and shoot‐to‐root ratio (S/R) (Figure 3; Table S5). Within the evergreen (Eg) group, LAR correlated positively only with LIF (*ρ* = 0.21, *p* < 0.05), while S/R showed strong positive associations with both POD_1_ (*ρ* = 0.29, *p* < 0.001) and LIF (*ρ* = 0.70, *p* < 0.001). In contrast, among deciduous (Dd) species, S/R correlated negatively with POD_1_ (*ρ* = −0.15, *p* = 0.0019) and LIF (*ρ* = −0.16, *p* = 0.0013), whereas LAR exhibited no significant associations.

## 4 Discussion

### 4.1 Evaluating Predictive Power of Ozone Metrics

Our results highlight POD_1_ as the most physiologically relevant O_3_ metric for predicting biomass reductions, effectively reflecting plant carbon allocation responses. In contrast, AOT40 showed weak correlations and limited predictive power, consistent with mechanistic expectations that O_3_ damage is better captured by stomatal flux than by ambient concentrations (Anav et al. 2016; De Marco et al. 2015; Paoletti et al. 2022) and in line with flux‐based recommendations of the CLRTAP Manual (Mills et al. 2017). LIF, which is strongly correlated with POD_1_, may provide complementary information (Manzini et al. 2023; Manzini, Garosi, et al. 2025; Manzini, Hoshika, et al. 2025), but its application to biomass responses did not provide better performances than POD_1_.

### 4.2 Enhanced Belowground Sensitivity: Distinct Root Biomass Responses to POD_1_

Our analyses demonstrate a clear and biologically consistent negative effect of O_3_ exposure, measured as POD_1_, on biomass allocation across all tree compartments. GEE revealed reductions in RTB, RTAB, and RTBB, showing the steepest decline, indicating that roots are disproportionately sensitive to O_3_ stress (Andersen 2003). This heightened belowground vulnerability has important ecological implications for resource acquisition, plant stability, and overall forest resilience under increasing O_3_ exposure.

Unlike aboveground tissues, which can mobilize resources for rapid leaf turnover and compensatory growth, root systems function as long‐term carbon sinks whose development is entirely dependent on sustained photosynthate supply from the canopy (Samuelson and Kelly 2001). When foliar O_3_ injury impairs carbon assimilation or triggers premature stomatal closure, the resulting photosynthetic deficit propagates belowground through reduced carbohydrate allocation, ultimately constraining root growth and function (Moura et al. 2021; Arab et al. 2022). This mechanism is consistent with the carbon starvation hypothesis (Hartmann 2015) and explains why RTBB declines precede and exceed those observed in aboveground compartments.

The disproportionate root sensitivity also reflects the absence of compensatory mechanisms available to shoots. While damaged leaves can be replaced within a growing season, root tissues accumulate chronic stress signals, including disrupted hormonal balance, impaired nutrient uptake, and reduced mycorrhizal associations, without the capacity for rapid regeneration (Arab et al. 2023; Agathokleous et al. 2020). Consequently, belowground biomass serves as an integrative indicator of cumulative O_3_ damage, capturing both direct metabolic disruption and indirect systemic effects that may remain invisible in aboveground assessments.

The steep RTBB decline observed across diverse species and exposure levels suggests that current O_3_ risk assessments may systematically underestimate long‐term impacts on tree stability, nutrient cycling, and ecosystem resilience. Given that S/R ratios influence drought tolerance, windthrow resistance, and belowground carbon sequestration (Mokany et al. 2006), the preferential impairment of root systems under O_3_ stress warrants urgent attention in climate‐forest interaction models and air quality policy frameworks.

The observation that biomass declines began even at the lowest observed POD_1_ exposures (ranging from 3.08 mmol m^−2^ in *A. unedo* to 69.5 mmol m^−2^ in *Populus* clone I‐214) indicates the absence of a true biological threshold below which O_3_ is harmless. This aligns with mounting evidence that chronic low‐level O_3_ exposure progressively damages photosynthetic machinery, impairing carbon assimilation (Benes et al. 1995; Dizengremel 2001) and ultimately reducing forest productivity at landscape scales. Modeling studies estimate net primary productivity reductions of 3%–16% across North America, up to 30% in Europe (Hoshika et al. 2015; Proietti et al. 2016), and approximately 9% in South Korea between 2001 and 2010 (Park et al. 2018). Our empirical data support these projections, revealing that belowground productivity losses likely exceed aboveground estimates, which could potentially amplify ecosystem‐level consequences.

### 4.3 POD_1_ Effects on Evergreen and Deciduous Relative Biomass

Evergreen and deciduous species exhibited distinct dose–response dynamics, reflecting contrasting adaptive strategies to O_3_ stress. Deciduous species showed greater overall sensitivity, experiencing more pronounced physiological and biochemical impairment under comparable O_3_ exposures.

Deciduous trees, characterized by rapid leaf expansion and seasonally high stomatal conductance, adopt an acquisitive strategy that maximizes photosynthesis and growth (Zhang et al. 2013) but concurrently increases O_3_ absorption during periods of elevated atmospheric concentrations. As a result, they exhibited steeper dose–response curves and faster biomass decline, particularly in RTBB, where even moderate POD_1_ levels led to reductions. These trends are consistent with field observations in the O_3_‐sensitive Oxford clone and the I‐214 hybrid clone (Carriero, Emiliani, et al. 2015; Carriero, Hoshika, et al. 2015; Hoshika, Pollastrini, et al. 2024), where chronic O_3_ exposure caused substantial reductions in both above‐ and belowground biomass. Stems and coarse roots were the most affected compartments, in line with the carbon starvation hypothesis (Hartmann 2015). According to this framework, foliar injury reduces photosynthetic carbon assimilation and can impair phloem transport, thereby limiting carbohydrate translocation to roots (King et al. 2005; Samuelson and Kelly 2001).

Over time, this limits belowground carbon allocation and drives RTBB decline even in the absence of VFI (Arab et al. 2022). Consequently, even slight increases above the CL_4_ threshold can significantly reduce deciduous forest biomass. Similar responses have been observed in subtropical forests, where deciduous species display earlier symptom onset and greater biomass losses than evergreens (Ren et al. 2011; Zhang et al. 2012), highlighting the global relevance of O_3_‐induced productivity decline.

Conversely, evergreen species demonstrated greater O_3_ tolerance across a broader exposure range, although their absolute biomass accumulation was lower. RTB and RTAB exhibited higher critical levels and required larger exposure increments to reach the 4% biomass loss threshold, while RTBB declined more gradually. The persistence of foliage over multiple seasons and tighter stomatal regulation reduce O_3_ uptake, but these traits alone cannot fully account for interspecific differences. O_3_ tolerance also depends on detoxification efficiency, repair capacity, and leaf structural attributes such as LMA (Calatayud et al. 2010; Feng, Uddling, et al. 2018).

High LMA values limit O_3_ diffusion through thicker tissues and smaller intercellular air spaces, providing mechanical protection against oxidative damage (Li et al. 2016). This structural resilience requires higher cumulative exposures to trigger measurable declines in RTAB and RTB, corroborating previous evidence that LMA is a key determinant of the superior O_3_ resilience of evergreens compared with deciduous trees (Feng, Uddling, et al. 2018; Manzini et al. 2023). However, the negative slopes observed for all evergreen compartments indicate that these defenses delay rather than prevent O_3_‐induced losses. Their buffering capacity may obscure chronic impairment, especially under long‐term, low‐level exposure typical of Mediterranean and temperate ecosystems, where physiological disruptions in carbon, water, and nutrient cycles can occur without visible injury (Grulke and Heath 2020).

These contrasting dose–response dynamics illustrate fundamental functional trade‐offs. Deciduous species are more vulnerable to acute, short‐term O_3_ peaks, as reflected by their relatively small Δ values (3.31, 5.70, 2.75 for RTB, RTAB, and RTBB), which indicate that even modest increases in POD_1_ rapidly trigger biomass reductions. In contrast, evergreens accumulate slower, chronic damage, with larger Δ values (10.78, 12.32, 8.40) showing that greater increases in POD_1_ are required to reach comparable biomass losses. Such divergent sensitivities, shaped by differences in foliar morphology, antioxidant capacity, and carbohydrate allocation, appear consistent across biogeographic regions. Tomlinson et al. (2013) showed that deciduous species allocate proportionally more non‐structural carbohydrates to roots than evergreens, supporting rapid canopy renewal but increasing vulnerability to O_3_‐induced carbon starvation when foliar injury disrupts assimilate flow. In conclusion, uniform O_3_ thresholds may therefore underestimate the vulnerability of deciduous forests while overlooking delayed degradation in evergreen systems. Root impairment, particularly in deciduous species, compromises ecosystem stability by reducing anchorage, water and nutrient uptake, and belowground carbon storage (McDowell et al. 2008; Mokany et al. 2006).

### 4.4 Comparison of Ozone Critical Levels

The assessment of tropospheric O_3_ impacts on vegetation has undergone methodological refinement over recent decades. Traditional concentration‐based metrics, such as AOT40, have progressively been supplemented or replaced by cumulative stomatal flux indices (phytotoxic ozone dose, POD), which provide a more realistic quantification of the ozone dose absorbed through stomata and integrate physiological responses under ambient environmental conditions. Despite these advances, the available literature remains fragmented, and cross‐study comparisons are challenging. A major source of heterogeneity lies in the choice of assessment endpoints. Most research has predominantly focused on visible foliar injury or on crown‐defoliation‐based critical levels (e.g., Marzuoli et al. 2019; Sicard et al. 2020, 2021), whereas fewer studies directly quantify critical thresholds for woody biomass reduction. Moreover, most experimental evidence originates from controlled environments, mainly open‐top chambers (OTC), where growth‐optimized conditions may not fully capture the complexity of field responses. In semi‐natural settings, plants experience multiple interacting stressors, resulting in variable and non‐linear responses (Hoshika, Agathokleous, et al. 2024). Further variability arises from the diversity of flux thresholds (Y) used in POD calculations (POD_0_, POD_0.5_, POD_1_, POD_1.6_, POD_2_, POD_3_) and from the criteria adopted to define biomass reduction thresholds (CL_2_, CL_4_, CL_5_). Currently, the CL_4_, representing a 4% biomass loss, is the threshold most commonly recommended by CLRTAP manual. Another emerging source of inconsistency concerns the definition of reference biomass, which varies depending on the plant compartments considered and on the methodologies employed to derive these values, including whether biomass is expressed in absolute terms or as relative measures in different formats.

Nevertheless, consistent patterns emerge. Deciduous species generally exhibit higher O_3_ sensitivity than evergreens, reflected in lower POD thresholds. In Mediterranean ecosystems, the CLRTAP mapping manual (Mills et al. 2017) reports CL_4_ thresholds for deciduous oaks of 14.0 mmol m^−2^ PLA for total biomass and 10.3 mmol m^−2^ PLA for root biomass. Marzuoli et al. (2018) confirmed similar values for *Q. robur* , *Q. faginea* Lam., and *Q. pyrenaica* Willd. under OTC conditions. Using a POD_1.6_ threshold, Calatayud et al. (2011) reported values around 16 mmol m^−2^ for the same species, further supporting the greater sensitivity of Mediterranean deciduous oaks.

For non‐Mediterranean deciduous species, Gerosa et al. (2015) estimated CL_4_–POD_1_ thresholds of 4 mmol m^−2^ for *Fagus sylvatica* L. and *Betula pendula* Roth. Based on Büker et al. (2015), who applied regression‐based dose–response models using POD_2_ and POD_3_, we estimated CL_4_ values for these species (e.g., 2.89 mmol m^−2^ for *F. sylvatica* and *B. pendula* , and 16.83 mmol m^−2^ for *Q. ilex and P. halepensis*), as they are not explicitly reported in the original publication. These examples illustrate how strongly the choice of POD threshold influences the estimated critical levels.

Hybrid poplars are particularly sensitive to O_3_. Gao et al. (2017) reported a CL_4_–POD_1_ of 5.27 mmol m^−2^ for a hybrid clone, while Hu et al. (2015) documented a CL_5_–POD_1_ of 6.1 mmol m^−2^ across five clones. Hoshika, Pollastrini, et al. (2024) confirmed that hybrid clones are highly sensitive (CL_4_–POD_1_ = 5.7 mmol m^−2^), whereas native, non‐hybrid poplars are more tolerant (CL_4_–POD_1_ = 10.3 mmol m^−2^). Among deciduous conifers, Japanese larch exhibits POD_1_ thresholds of 4.19–4.31 mmol m^−2^ (Hoshika et al. 2020), clearly below typical recommended values for non‐Mediterranean species, highlighting its particular vulnerability.

Water availability strongly modulates O_3_ effects. In a FACE experiment, Hoshika et al. (2018) reported a CL_5_–POD_0.5_ of 3.5 mmol m^−2^ for *Q. robur* subjected to combined O_3_ and drought stress, compared with 6.8 mmol m^−2^ in less sensitive species, demonstrating the integrative capacity of stomatal flux‐based metrics.

Evergreen Mediterranean species typically show greater resilience to O_3_. The CLRTAP Mapping Manual indicates that *Q. ilex* has a CL_4_–POD_1_ of 47.3 mmol m^−2^ PLA for aboveground biomass. Calatayud et al. (2011) reported a CL_5_–POD_5_ of approximately 53 mmol m^−2^ for *Q. ilex* (increasing to 72 mmol m^−2^ when considering the full 12‐month leaf‐activity period), providing further support for the high resistance of this species under elevated ozone exposure. In contrast, Gerosa et al. (2015) proposed a much lower critical limit (CL_4_–POD_1_ = 7 mmol m^−2^ for total biomass). Furthermore, Büker et al. (2015) estimated CL_4_–POD_1_ at 16.83 mmol m^−2^ for *Q. ilex* and derived comparable thresholds for *P. halepensis* , underscoring the substantial variability in critical levels introduced by differences in POD threshold selection, ozone metric, and biomass endpoint.

Our research provides the first comprehensive comparison of evergreen and deciduous species. For the deciduous group, the critical levels we determined are CL_4_–POD_1_ = 10.77 mmol m^−2^ (total biomass) and 13.16 mmol m^−2^ (aboveground biomass). These values are consistent with those reported by Marzuoli et al. (2018) and exceed the conservative thresholds established for beech and birch. For evergreens, CL_4_–POD_1_ ranges from 11.48 to 15.40 mmol m^−2^ depending on the biomass compartment, reflecting intra‐group variability driven by leaf longevity and carbon allocation strategies. These results underline the importance of adopting functional‐group‐specific thresholds for O_3_ risk assessment and for informing forest‐management strategies. Although CL_4_–POD_1_ values were derived at the functional‐group level, the potential influence of individual species was explicitly assessed using a leave‐one‐species‐out (LOSO) sensitivity analysis. This analysis showed that CL_4_ estimates for deciduous species were generally stable across iterations, indicating that group‐level responses were not disproportionately driven by any single taxon. While exclusion of the O_3_‐sensitive Oxford poplar clone resulted in slightly higher CL_4_ estimates, overall variability remained limited, supporting the robustness of the deciduous group response and the more pronounced O_3_ sensitivity of this clone. In contrast, evergreen species exhibited notable interspecific variability, consistent with their broader functional and physiological diversity. In particular, the pronounced O_3_ sensitivity of *A. unedo* contributed disproportionately to the dispersion of CL_4_ estimates observed within the evergreen group.

Previous studies classified *A. unedo* as relatively O_3_‐tolerant based on limited visible injury, stable stomatal conductance, and enhanced antioxidant capacity under elevated AOT40 (Nali et al. 2004; Paoletti 2005). However, such tolerance was primarily inferred from short‐term physiological and foliar responses. Our flux‐based biomass analysis indicates that, despite its low cumulative POD_1_ and limited foliar injury reported under FACE conditions (Manzini et al. 2023), *A. unedo* shows comparatively stronger biomass responses per unit of O_3_ uptake. This suggests that physiological tolerance does not necessarily translate into long‐term growth resilience when O_3_ effects are evaluated using integrative, growth‐related measurements. An explanation could be that chronic, sub‐lethal O_3_ exposure may preferentially affect carbon allocation and growth processes in this species, even in the absence of marked stomatal limitation or visible injury.

Despite the heterogeneity observed between deciduous and evergreen species, the relative contrast in O_3_ sensitivity between the two groups was preserved across all biomass compartments, supporting the use of leaf habit‐based aggregation for O_3_ risk assessment under the FACE experimental conditions considered, while acknowledging the inherent limitations of this experimental approach.

### 4.5 Ecophysiological Trade‐Offs in Biomass Allocation Across O_3_ Gradients

The correlation analysis revealed consistent and physiologically meaningful patterns in biomass allocation under O_3_ exposure. In deciduous (Dd) species, increasing O_3_ tended to reduce the efficiency of carbon allocation to shoots relative to roots (lower S/R), whereas in evergreen (Eg) species it promoted a coordinated enhancement of both leaf area and shoot investment (higher LAR and S/R). These contrasting responses highlight divergent carbon balance strategies under O_3_ stress.

This pattern was particularly evident in deciduous species, where the shoot‐to‐root ratio (S/R) decreased with increasing POD_1_ or LIF. The shifts reflect the coincidence of exposure timing with the short lifespan of leaves. In fact, several deciduous species exhibit accelerated leaf senescence as a response to O_3_ stress (Pell et al. 1997), indicating that leaf turnover can be a strategy to maintain root function, with roots being prioritized to ensure water and nutrient uptake, while leaves suffer the greatest damage. The genotype‐specific patterns reinforce this interpretation, revealing that leaf injury severity correlates with phenolic composition and antioxidant cost, and that root investment is modulated by both defense strategy and stomatal behavior (Yamaji et al. 2003). These effects vary by plant species and individual genotype, as shown by Yamaji et al. (2003), who observed divergent S/R responses among *Betula pendula* Roth. clones under elevated O_3_ exposures. Most of the clones exhibited a decrease in S/R consistent with carbon reallocation to roots and stomatal closure. This high variability is evident not only at the species level but also among ecotypes. Studies on different ecotypes allow us to observe variability in phenotypic responses, reflecting both plasticity and local genetic adaptations. For example, Moura et al. (2021) reported that, in *Moringa oleifera* Lam., ecotype‐level analysis under elevated O_3_ showed reductions in leaf biomass across all ecotypes, with variable adjustments in the S/R ratio. Ecotypes with higher stomatal conductance exhibited greater shoot reductions relative to roots, suggesting carbon reallocation as a compensatory strategy. These findings highlight the role of genotypic variability in stomatal regulation and carbon allocation for O_3_ tolerance, as previously observed in temperate species.

In evergreen species, the S/R increased with increasing POD_1_ or LIF, suggesting that plants reallocate biomass toward aboveground compartments to preserve overall functionality, while structurally resilient and long‐lived leaves tolerate stress without drastic changes in relative share. This is consistent with the observation that Mediterranean evergreen species, already adapted to oxidative stressors such as drought and high radiation, exhibit enhanced tolerance to O_3_ through morpho‐anatomical traits and conservative stomatal behavior (Paoletti 2007). Furthermore, LIF underscores structural vulnerability, as species exhibiting low LMA and elevated stomatal flux demonstrated heightened LIF values, signifying diminished defensive capacity and an increased likelihood of observable foliar damage. In contrast, species characterized by thick, dense leaves and low LIF effectively manage oxidative stress through a combination of avoidance and tolerance mechanisms (Manzini et al. 2023).

Differences between evergreen and deciduous species reflect adaptive strategies related to leaf lifespan and structure, phenological plasticity, and the need to preserve root and structural functionality under O_3_ stress (Paoletti 2007). These mechanisms illustrate how plants optimize resource distribution to maintain vital functions rather than maximize absolute growth.

### 4.6 LMA of *Cupressus sempervirens* Clone 2546 and 3375

LMA values observed in *C. sempervirens* clones 2546 and 3375 (424.82 and 434.08 g/m^2^, respectively) were substantially higher than those reported in previous studies. For instance, Scartazza et al. (2025) documented an average of 318.5 ± 12.4 g/m^2^, while (Farahat and Linderholm 2012) reported values between 21.6 and 35.8 g/m^2^ in Egyptian forests. These discrepancies likely reflect the species' phenotypic plasticity (Zorer et al. 2014) and differences in experimental conditions. Unlike earlier studies conducted under natural Mediterranean stress conditions, our clones were grown under controlled O_3_ exposure without water limitation. While drought‐induced leaf anatomical changes are well documented in broadleaf species (Yavas et al. 2024), conifers like *C. sempervirens* possess inherently thick, structurally reinforced needles and may rely on alternative adaptive mechanisms. The response of the two clones to increasing O_3_ exposure revealed subtle intraspecific genetic variability. Both showed progressive increases in POD_1_, with nearly identical LIF values under the 2.0 × AA treatment (0.047 mmol/g for clone 2546 and 0.046 mmol/g for clone 3375). Despite similar LMA and uptake metrics, these minor differences may reflect distinct metabolic or defensive strategies. *C. sempervirens* exhibits a conservative foliar strategy aligned with the slow‐return end of the leaf economics spectrum (Reich 2014; Wright et al. 2004). This position on the spectrum indicates species that invest heavily in leaf construction (high LMA) and prioritize durability and resource conservation over rapid growth. Such leaves typically have low nutrient concentrations and modest photosynthetic rates, but their long lifespan ensures structural resilience and sustained function under stress. This strategy is well adapted to Mediterranean environments, where water and nutrients are scarce and stressors such as O_3_ pollution are frequent. Photosynthetic activity peaks at about 2 years of needle age, then declines due to reduced photochemical capacity and loss of key polypeptides (La Porta et al. 2006).

## 5 Conclusions

This study confirmed that flux‐based metrics represent biologically more meaningful indicators of O_3_ effects on biomass compared to concentration‐based indices. Among the analyzed metrics, POD_1_ proved to be robust and physiologically relevant in predicting total, aboveground, and belowground biomass reductions relative to theoretical pre‐industrial values. Indices such as LIF represent a promising alternative to explore further, by developing appropriate and specific biomass reduction thresholds for this index, considering the variability between deciduous and evergreen species and morpho‐physiological differences.

Deciduous species showed steeper dose–response curves and lower critical levels for a 4% biomass reduction (CL_4_ = 10.77 mmol m^−2^ for RTB, 13.16 mmol m^−2^ for RTAB, and 10.21 mmol m^−2^ for RTBB), indicating greater sensitivity to O_3_, while evergreens demonstrated superior tolerance (CL_4_ = 13.86 mmol m^−2^ for RTB, 15.40 mmol m^−2^ for RTAB, and 11.48 mmol m^−2^ for RTBB) due to high LMA values and conservative stomatal control, although still recording potential biomass losses under chronic exposures. The Δ values, corresponding to the variation in POD_1_ required to reach the critical threshold of a 4% biomass reduction (CL_4_), highlight that deciduous species, with relatively small Δ values (3.31, 5.70, 2.75 for RTB, RTAB, and RTBB), reach the critical effect rapidly even with modest changes in POD_1_, indicating, as with similar values in evergreens, a high sensitivity. In contrast, evergreen species, with larger Δ values (10.78, 12.32, 8.40), require greater changes in POD_1_ to achieve the same reduction, confirming a higher tolerance, while still exhibiting potential biomass losses under chronic exposures. In this sense, Δ provides a quantitative indication of vulnerability, integrating both the magnitude and the rate of response of different species to O_3_. Root biomass proved to be the most sensitive compartment, supporting the “carbon starvation” hypothesis: leaf damage limits carbon flow to roots, compromising growth, nutrient uptake, and ecosystem stability. Analysis of biomass partitioning revealed relative reductions in leaves, reallocation toward roots, and proportional increases in stems, consistent with temporary compensatory adaptive mechanisms. Differences between evergreen and deciduous leaves reflect strategies related to lifespan, structure, and phenological plasticity, as well as the balance between chemical defense, stomatal conductance, and carbon allocation.

Based on controlled experimental conditions and a limited number of species and clones, with broader inferences primarily framed around leaf habit, the present study should be interpreted as a contextualized snapshot rather than a comprehensive representation of forest‐scale O_3_ responses. In particular, the results are constrained by the spatial and temporal scope of a single‐site FACE experiment and by the treatment of O_3_ as an isolated stressor, without explicitly accounting for interactions with co‐occurring environmental drivers such as temperature, drought, nutrient availability, or elevated CO_2_. Future research should therefore expand the analysis to a wider range of species, genotypes, and functional strategies, refine and validate biomass reduction thresholds for alternative flux‐based indices such as LIF, and explicitly embed O_3_ uptake within multi‐stressor analytical frameworks. Such integrative approaches will be essential to improve predictions of critical levels, disentangle trade‐offs among plant compartments and carbon allocation pathways, and enhance the robustness and ecological realism of O_3_ impact models across Mediterranean, temperate, boreal, and tropical forest ecosystems.

## Author Contributions

**Annesha Ghosh:** conceptualization, methodology, data curation, writing – original draft. **Andrea Viviano:** conceptualization, validation, formal analysis, investigation, data curation, writing – original draft, writing – review and editing, visualization. **Yasutomo Hoshika:** conceptualization, resources, data curation, writing – review and editing, supervision, project administration, funding acquisition. **Elena Marra:** investigation, writing – review and editing. **Jacopo Manzini:** data curation, validation, investigation, writing – review and editing. **Cesare Garosi:** investigation, writing – review and editing. **Elena Paoletti:** supervision, project administration, funding acquisition, writing – review and editing. **Matheus Casarini Siqueira:** investigation. **Barbara B. Moura:** methodology, data curation, validation, investigation, writing – review and editing, visualization.

## Funding

This work was supported by the Italian Ministry of University and Research (MUR) within the framework of the National Recovery and Resilience Plan (NRRP), funded by the European Union – NextGenerationEU, Mission 4, Component 2: Investment 1.4, Call for tender No. 3138 of 16 December 2021 (rectified by Decree No. 3175 of 18 December 2021), Award Number Project code CN_00000033, Concession Decree No. 1034 of 17 June 2022, CUP B83C22002930006, Project title “National Biodiversity Future Center – NBFC” (Spoke 5); Investment 3.1 “Fund for the realisation of an integrated system of research and innovation infrastructures” ‐ Project IR0000032 – ITINERIS ‐ Italian Integrated Environmental Research Infrastructures System ‐ CUP B53C22002150006; and Department of Science and Technology, Ministry of Science and Technology, India. INSPIRE division India Faculty Fellowship (IFA22‐LSPA 154, 10.13039/50110001409).

## Conflicts of Interest

The authors declare no conflicts of interest.

## Supporting information


**Data S1:** gcb70728‐sup‐0001‐supinfo.docx.

## Data Availability

The data that support the findings of this study are openly available in Mendeley at https://data.mendeley.com/datasets/92x6gxj3mw/2, reference number https://doi.org/10.17632/92x6gxj3mw.2.
