# Supplementary material for: Tree Biomass Sensitivity to Ozone Exposure: Insights From a Decade of Free‐Air Experiments
Source: Glob Chang Biol. 2026 Jan 29;32(2):e70728. doi: 10.1111/gcb.70728 (PMC12853240; doi:10.1111/gcb.70728)
Supplement: Supplementary file 1 — Data S1: gcb70728‐sup‐0001‐supinfo.docx. [file GCB-32-e70728-s001.docx]

**Tree biomass sensitivity to ozone exposure: Insights from a decade of Free-Air experiments**

[Annesha Ghosh, Andrea Viviano, Elena Paoletti, Yasutomo Hoshika, Elena Marra, Jacopo Manzini](https://scholar.google.com/citations?user=05mXK5gAAAAJ&hl=en) [, Cesare Garosi,](https://scholar.google.com/citations?user=05mXK5gAAAAJ&hl=en)  Matheus Casarini Siqueira, [Barbara B. Moura](https://scholar.google.com/citations?user=05mXK5gAAAAJ&hl=en)

*Supplementary material*

**- Figure section -**


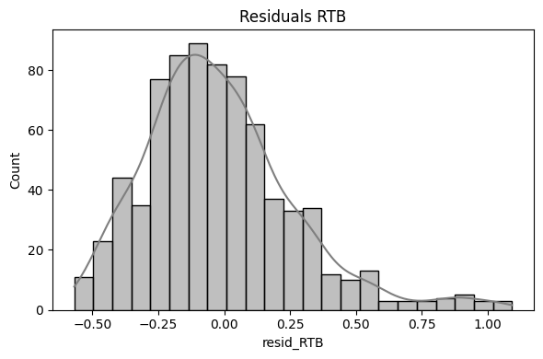

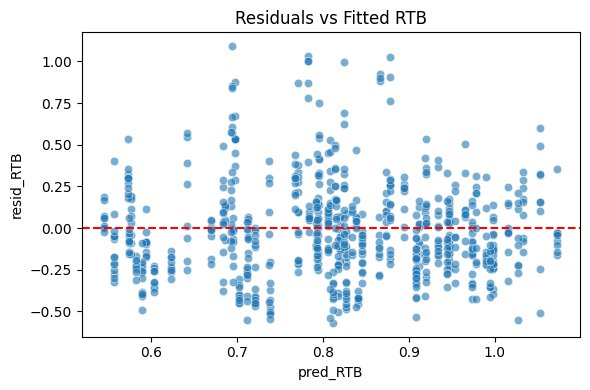

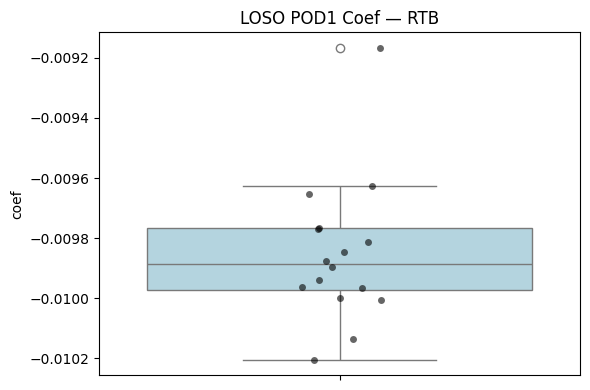

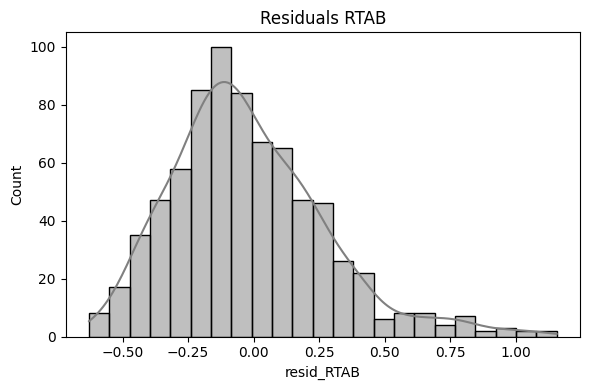

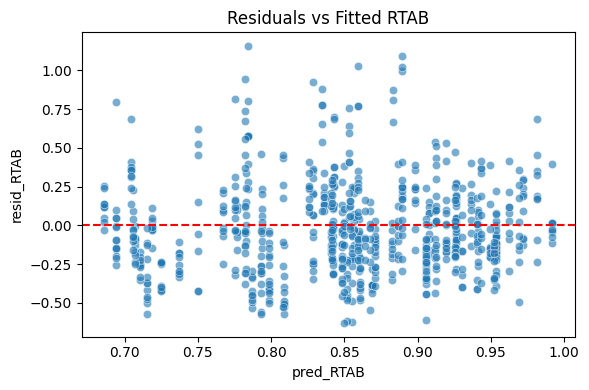

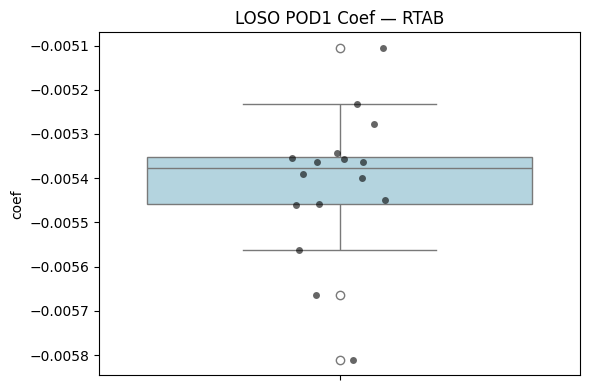

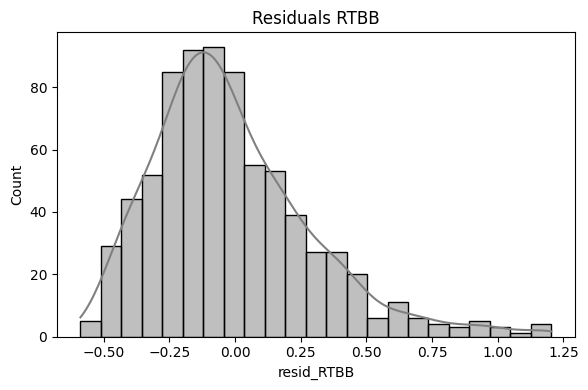

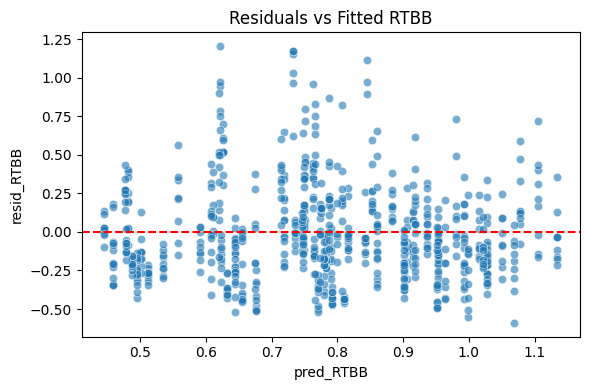

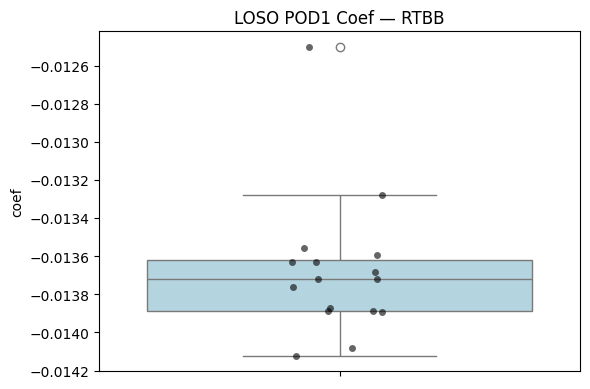


Figure S1: Diagnostic plots for three regression models ((Relative total biomass -RTB, Relative biomass above ground - RTAB, and below ground - RTBB) assessing residual behavior and coefficient stability. Left column: histograms of residuals show asymmetric but coherent distributions, consistent with the Gamma-log specification. Middle column: residuals plotted against fitted values reveal no systematic patterns or heteroscedasticity, supporting model adequacy. Right column: boxplots of POD1 coefficients from Leave-One-Subject-Out cross-validation indicate stable and consistently negative effects across models, confirming robustness of the estimated associations.

- **Table section -**

**Table S1.** Target species and leaf habit (evergreen/deciduous), O_3_ exposure period, and year of sample collection, Ref= references

| **Species** | **Leaf habit** | **Period of exposure** | **Year of treatment** | **Ref.** |
| --- | --- | --- | --- | --- |
| *Arbutus unedo* | Evergreen | June 12 – October 15 | 2017 | Meschini et al. (2023) |
| *Carpinus betulus* | Deciduous | May 23 – October 16 | 2023 | Unpublished |
| *Cupressus sempervirens clones 2546* | Evergreen | May 01 – October 01 | 2019,2020 | Manzini et al. (2025a) |
| *Cupressus sempervirens clones 3375* | Evergreen | May 01 – October 01 | 2019,2020 | Manzini et al. (2025a) |
| *Ostrya carpinifolia* | Deciduous | May 23 – October 16 | 2023 | Unpublished |
| *Oxford poplar* | Deciduous | May 01 – October 01 | 2016 | Zhang et al. (2018); Hoshika et al. (2024) |
| *Phyllirea angustifolia* | Evergreen | May 01 – October 31 | 2018 | Pellegrini et al. (2021) |
| *Pinus halepensis* | Evergreen | May 15 – October 20 | 2019 | Hoshika et al. (2023) |
| *Pinus pinea* | Evergreen | May 15 – October 20 | 2019 | Hoshika et al. (2023) |
| *Poplar i-214 clone* | Deciduous | May 01 – October 01 | 2020 | Hoshika et al. (2024) |
| *Quercus ilex* | Evergreen | June 01 – October 15 | 2015 | Hoshika et al. (2018) |
| *Quercus pubescens* | Deciduous | June 01 – October 15 | 2015 | Hoshika et al. (2018) |
| *Quercus robur* | Deciduous | June 01 – October 15 | 2015 | Hoshika et al. (2018) |
| *Robinia pseudoacacia* | Deciduous | May 01 – October 01 | 2024 | Hoshika et al. (2025) In press |
| *Schinus rebinthifolia* | Evergreen | May 01 – October 01 | 2024 | Casarini et al. (2025) In press |
| *Vaccinium myrtillus* | Deciduous | May 01 – October 01 | 2018 | Hoshika et al. (2022); Manzini et al. (2023) |

**Table S2a.** Mean values of ozone exposure indices: POD₁ (mmol/m² PLA), LIF (mmol/g), AOT40 (ppm h), and M24 (ppb)—for each target species across the three ozone treatments where AA is ozone level in ambient air.

| **Species** | **Treatment** | **POD_1_** | **LIF** | **AOT40** | **M24** | |
| --- | --- | --- | --- | --- | --- | --- |
| *Arbutus unedo* | AA | 3.08 | 0.0234 | 23.2 | 40.2 | |
| *Arbutus unedo* | 1.5xAA | 5.04 | 0.0383 | 44.48 | 53.4 | |
| *Arbutus unedo* | 2.0xAA | 6.88 | 0.0524 | 63.54 | 65.8 | |
| *Carpinus betulus* | AA | 12.89 | 0.1359 | 16.0 | 36.8 | |
| *Carpinus betulus* | 1.5xAA | 19.6 | 0.2067 | 40.11 | 49.8 | |
| *Carpinus betulus* | 2.0xAA | 29.56 | 0.3118 | 78.24 | 66.2 | |
| *Cupressus 2546* | AA | 10.39 | 0.0245 | 48.7 | 38.51 | |
| *Cupressus 2546* | 1.5xAA | 15.82 | 0.0372 | 112.98 | 54.7 | |
| *Cupressus 2546* | 2.0xAA | 19.96 | 0.047 | 176.39 | 71.5 | |
| *Cupressus 3375* | AA | 10.39 | 0.0239 | 48.7 | 38.51 | |
| *Cupressus 3375* | 1.5xAA | 15.82 | 0.0386 | 112.98 | 54.7 | |
| *Cupressus 3375* | 2.0xAA | 19.96 | 0.046 | 176.39 | 71.5 | |
| *Ostrya carpinifolia* | AA | 8.72 | 0.0795 | 16.0 | 36.8 | |
| *Ostrya carpinifolia* | 1.5xAA | 13.84 | 0.1262 | 40.11 | 49.8 | |
| *Ostrya carpinifolia* | 2.0xAA | 21.51 | 0.1962 | 78.24 | 66.2 | |
| *Oxford* | AA | 33.63 | 0.3656 | 18.22 | 36.99 | |
| *Oxford* | 1.5xAA | 43.51 | 0.1998 | 45.72 | 52.06 | |
| *Oxford* | 2.0xAA | 61.24 | 0.708 | 81.26 | 72.96 | |
| *Phyllirea angustifolia* | AA | 11.21 | 0.098 | 18.91 | 36.2 | |
| *Phyllirea angustifolia* | 1.5xAA | 18.8 | 0.1003 | 49.71 | 54.7 | |
| *Phyllirea angustifolia* | 2.0xAA | 24.89 | 0.1327 | 77.31 | 67.2 |  |
| *Pinus halepensis* | AA | 17.15 | 0.081 | 23.93 | 38.0 | |
| *Pinus halepensis* | 1.5xAA | 27.3 | 0.1289 | 61.24 | 56.1 | |
| *Pinus halepensis* | 2.0xAA | 33.53 | 0.1583 | 82.62 | 68.5 | |
| *Pinus pinea* | AA | 18.62 | 0.0717 | 23.93 | 38.0 | |
| *Pinus pinea* | 1.5xAA | 29.83 | 0.1149 | 61.24 | 56.1 | |
| *Pinus pinea* | 2.0xAA | 36.52 | 0.1406 | 82.62 | 68.5 | |
| *Poplar i-214 clone* | AA | 33.67 | 0.3855 | 21.27 | 36.99 | |
| *Poplar i-214 clone* | 1.5xAA | 48.81 | 0.5589 | 50.69 | 52.06 | |
| *Poplar i-214 clone* | 2.0xAA | 69.5 | 0.7958 | 92.9 | 72.96 | |
| *Quercus ilex* | AA | 10.77 | 0.0648 | 17.07 | 35.1 | |
| *Quercus ilex* | 1.2xAA | 12.93 | 0.0778 | 25.92 | 43.0 | |
| *Quercus ilex* | 1.4xAA | 15.04 | 0.0904 | 34.56 | 49.0 | |
| *Quercus pubescens* | AA | 24.0 | 0.2843 | 17.07 | 35.1 | |
| *Quercus pubescens* | 1.2xAA | 28.08 | 0.3327 | 25.92 | 43.0 | |
| *Quercus pubescens* | 1.4xAA | 31.83 | 0.3771 | 34.56 | 49.0 | |
| *Quercus robur* | AA | 23.44 | 0.3437 | 17.07 | 35.1 | |
| *Quercus robur* | 1.2xAA | 23.44 | 0.3437 | 25.92 | 43.0 | |
| *Quercus robur* | 1.4xAA | 31.11 | 0.4561 | 34.56 | 49.0 | |
| *Robinia pseudoacacia* | AA | 31.0 | 0.440 | 21.02 | 40.8 | |
| *Robinia pseudoacacia* | 1.5xAA | 41.17 | 0.585 | 39.36 | 52.2 | |
| *Robinia pseudoacacia* | 2.0xAA | 55.11 | 0.783 | 65.87 | 66.9 | |
| *Schinus terebinthifolia* | AA | 28.7 | 0.293 | 16.38 | 39.9 | |
| *Schinus terebinthifolia* | 1.5xAA | 37.08 | 0.379 | 27.88 | 53 | |
| *Schinus terebinthifolia* | 2.0xAA | 48.48 | 0.495 | 45.86 | 69.3 | |
| *Vaccinium myrtillus* | AA | 7.46 | 0.098 | 13.9 | 38.4 | |
| *Vaccinium myrtillus* | 1.5xAA | 12.49 | 0.165 | 35.28 | 57.6 | |
| *Vaccinium myrtillus* | 2.0xAA | 16.04 | 0.211 | 52.66 | 76.6 | |

**Table S2b**. Leaf Mass per Area (LMA) values (g/m²) for selected species and clones, with corresponding literature references or indication of original data (*Ex-novo* data). The table includes a range of broadleaf and conifer species commonly studied for ecophysiological traits related to ozone exposure.

| Species | LMA (g/m^2^) | Reference |
| --- | --- | --- |
| *‘Oxford’ poplar* | 86.50 | Zhang et al. (2018); Hoshika et al. (2024) |
| *Arbutus unedo* | 131.3 | Manzini et al. 2025b |
| *Carpinus betulus* | 94.80 | Pagano et al. (2024) |
| *Cupressus* 2546 | 424.82 | *Ex-novo* data |
| *Cupressus* 3375 | 434.08 | *Ex-novo* data |
| *Ostrya carpinifolia* | 109.6 | Pagano et al. (2024) |
| *Phyllirea angustifolia* | 187.50 | Pellegrini et al. (2021) |
| *Pinus halepensis* | 211.80 | Hoshika et al. (2023) |
| *Pinus pinea* | 259.60 | Hoshika et al. (2023) |
| *Poplar i-214 clone* | 87.33 | Manzini et al. (2023) |
| *Quercus ilex* | 166.20 | Hoshika et al. (2018) |
| *Quercus pubescens* | 84.4 | Hoshika et al. (2018) |
| *Quercus robur* | 68.20 | Hoshika et al. (2018) |
| *Robinia pseudoacacia* | 70.4 | Hoshika et al. (2025) In press |
| *Schinus terebinthifolia* | 97.9 | *Ex-novo* data |
| *Vaccinium myrtillus* | 75.9 | Hoshika et al. (2022); Manzini et al. (2023) |

**Table S3.** Relative biomass referenced to the preindustrial era and associated equations. Bref = reference biomass at 20 ppb in the preindustrial era, used to calculate relative biomass (total - TB, aboveground TAB, and belowground TBB biomass).

| **Species** | **Biomass type** | **RBref** |
| --- | --- | --- |
| *Arbutus unedo* | TAB | 213.59 |
| *Arbutus unedo* | TBB | 62.260 |
| *Arbutus unedo* | TB | 272.444 |
| *Carpinus betulus* | TAB | 166.894 |
| *Carpinus betulus* | TBB | 72.947 |
| *Carpinus betulus* | TB | 239.836 |
| *Cupressus 2546* | TAB | 269.142 |
| *Cupressus 2546* | TBB | 126.722 |
| *Cupressus 2546* | TB | 395.864 |
| *Cupressus 3375* | TAB | 317.512 |
| *Cupressus 3375* | TBB | 146.87 |
| *Cupressus 3375* | TB | 464.384 |
| *Ostrya carpinifolia* | TAB | 118.782 |
| *Ostrya carpinifolia* | TBB | 63.713 |
| *Ostrya carpinifolia* | TB | 182.494 |
| *Oxford* | TAB | 27.636 |
| *Oxford* | TBB | 38.826 |
| *Oxford* | TB | 66.462 |
| *Phyllirea angustifolia* | TAB | 438.91 |
| *Phyllirea angustifolia* | TBB | 123.612 |
| *Phyllirea angustifolia* | TB | 562.522 |
| *Pinus halepensis* | TAB | 215.908 |
| *Pinus halepensis* | TBB | 43.127 |
| *Pinus halepensis* | TB | 259.03 |
| *Pinus pinea* | TAB | 38.3998 |
| *Pinus pinea* | TBB | 15.563 |
| *Pinus pinea* | TB | 53.963 |
| *Poplar i-214 clone* | TAB | 28.945 |
| *Poplar i-214 clone* | TBB | 47.471 |
| *Poplar i-214 clone* | TB | 76.415 |
| *Quercus ilex* | TAB | 11.306 |
| *Quercus ilex* | TBB | 12.932 |
| *Quercus ilex* | TB | 24.238 |
| *Quercus pubescens* | TAB | 13.491 |
| *Quercus pubescens* | TBB | 21.039 |
| *Quercus pubescens* | TB | 34.531 |
| *Quercus robur* | TAB | 14.074 |
| *Quercus robur* | TBB | 27.803 |
| *Quercus robur* | TB | 41.877 |
| *Robinia pseudoacacia* | TAB | 25.118 |
| *Robinia pseudoacacia* | TBB | 16.135 |
| *Robinia pseudoacacia* | TB | 41.25 |
| *Schinus terebinthifolia* | TAB | 98.39 |
| *Schinus terebinthifolia* | TBB | 50.57 |
| *Schinus terebinthifolia* | TB | 148.96 |
| *Vaccinium myrtillus* | TAB | 121.33 |
| *Vaccinium myrtillus* | TBB | 33.68 |
| *Vaccinium myrtillus* | TB | 155.01 |

**Table S4.** Summary of model specifications and coefficient estimates from GEE Gamma-log regressions. The table reports the response variable, predictor, distribution family, link function, number of clusters, estimated coefficient for POD_1_, standard error, z-statistic, p-value, 95% confidence interval, and model scale parameter (dispersion parameter of the Gamma model, reflecting residual variability).

| \| **Response** \| **Predictor** \| **Family** \| **Link** \| **Clusters** \| **Coef (POD_1_)** \| **Std. err** \| **z** \| **p-value** \| **95% confidence interval** \| **scale** \| \| --- \| --- \| --- \| --- \| --- \| --- \| --- \| --- \| --- \| --- \| --- \| \| RTB \| POD1 \| Gamma \| Log \| 16 \| −0.0098 \| 0.001 \| −9.926 \| <0.001 \| [−0.012, −0.008] \| 0.135 \| \| RTAB \| POD1 \| Gamma \| Log \| 16 \| −0.0054 \| 0.000 \| −11.720 \| <0.001 \| [−0.006, −0.004] \| 0.126 \| \| RTBB \| POD1 \| Gamma \| Log \| 16 \| −0.0136 \| 0.002 \| −8.332 \| <0.001 \| [−0.017, −0.010] \| 0.179 \|   **Table S5.** Distance correlation (*dcor*) and Spearman’s rank correlation coefficients (*rₛ*) between ozone indices (POD₁ and LIF) and biomass allocation indices, Leaf Area Ratio (LAR), and the root-to-shoot ratio (S/R) in evergreen (Eg), deciduous (Dd) species, *dcor* values represent the nonparametric distance correlation between variables, while *rₛ* represents the direction and strength of monotonic association.   \| **Group** \| **Independent variable** \| **Dependent variable** \| **Distance correlation (*dcor*)** \| **Spearman’s *rₛ*** \| ***p*-value** \| \| --- \| --- \| --- \| --- \| --- \| --- \| \| **Eg** \| POD_1_ \| S/R \| 0.40 \| 0.29 \| <0.001 \| \|  \| LIF \| LAR \| 0.32 \| 0.21. \| <0.05 \| \|  \| LIF \| S/R \| 0.70 \| 0.70 \| <0.001 \| \| **Dd** \| POD_1_ \| S/R \| 0.36 \| -0.15 \| <0.05 \| \|  \| LIF \| S/R \| 0.37 \| -0.16 \| <0.05 \| |
| --- | --- | --- | --- | --- | --- | --- | --- | --- | --- | --- | --- | --- | --- | --- | --- | --- | --- | --- | --- | --- | --- | --- | --- | --- | --- | --- | --- | --- | --- | --- | --- | --- | --- | --- | --- | --- | --- | --- | --- | --- | --- | --- | --- | --- | --- | --- | --- | --- | --- | --- | --- | --- | --- | --- | --- | --- | --- | --- | --- | --- | --- | --- | --- | --- | --- | --- | --- | --- | --- | --- | --- | --- | --- | --- | --- | --- | --- | --- | --- | --- |

- **Section 1: Cypress O_3_ indices and Leaf Mass per Area detection -**

LMA values were comparable between the two *Cupressus sempervirens* clones. Clone 2546 exhibited an average LMA of 424.82 g/m², while clone 3375 showed a slightly higher value of 434.08 g/m².
 Ozone exposure indices varied across treatments but remained similar between the two clones. For clone 2546, POD₁ values increased from 10.39 mmol m⁻² in AA, to 15.82 mmol m⁻² under 1.5×AA, and reached 19.96 mmol m⁻² under 2.0×AA conditions. Corresponding LIF (mmol/g) values were 0.0222, 0.0440, and 0.0470, while AOT40 increased from 48.70 to 176.39 ppm·h across the same treatments. Clone 3375 exhibited identical POD₁ and AOT40 values across treatments (10.39, 15.82, and 19.96 mmol m⁻² for POD₁; 48.70, 112.98, and 176.39 ppm·h for AOT40), while its LIF values showed greater variation, rising from 0.0230 (AA) to 0.0386 (1.5×AA) and peaking at 0.2953 under 2.0×AA (Tables S2a,b).

**References**
1. Hoshika, Y., Moura, B., Paoletti, E. (2018). Ozone risk assessment in three oak species as affected by soil water availability. Environmental Science and Pollution Research, 25, 8125-8136.

2. Manzini, J., Hoshika, Y., Danti, R., Moura, B. B., Paoletti, E., & Della Rocca, G. (2025a). Ozone risk assessment of common cypress (*Cupressus sempervirens* L.) clones and effects of Seiridium cardinale infection. journal of environmental sciences, 151, 441-453.

3. Hoshika, Y., Pollastrini, M., Marzuoli, R., Gerosa, G., Marra, E., Moura, B. B., ... & Paoletti, E. (2024). Unraveling the difference of sensitivity to ozone between non-hybrid native poplar and hybrid poplar clones: A flux-based dose-response analysis. Environmental Pollution, 358, 124524.

4. Pellegrini, E., Cotrozzi, L., Neri, L., Baraldi, R., Carrari, E., Nali, C., ... & Hoshika, Y. (2021). Stress markers and physiochemical responses of the Mediterranean shrub Phillyrea angustifolia under current and future drought and ozone scenarios. Environmental Research, 201, 111615.

5. Hoshika, Y., Cotrozzi, L., Gavrichkova, O., Nali, C., Pellegrini, E., Scartazza, A., Paoletti, E. (2023). Functional responses of two Mediterranean pine species in an ozone Free-Air Controlled Exposure (FACE) experiment. Tree Physiology. 43, 1548–1561

6. Meschini, R., Paoletti, E., Hoshika, Y., Sideri-Manoka, Z. A., Dell'Orso, A., Magni, G., & Kuzminsky, E. (2023). Comet assay as an early predictor tool to detect ozone enhanced sensitivity of vegetation in a free-air controlled long-term exposure. Plant Stress, 10, 100236.

7. Manzini, J., Hoshika, Y., Moura, B. B., & Paoletti, E. (2023). Exploring a new O3 index as a proxy for the avoidance/tolerance capacity of forest species to tolerate O3 injury. Forests, 14(5), 901.

8.Zhang, L., Hoshika, Y., Carrari, E., Cotrozzi, L., Pellegrini, E., Paoletti, E. (2018). Effects of nitrogen and phosphorus imbalance on photosynthetic traits of poplar Oxford clone under ozone pollution. *Journal of Plant Research,* *131*, 915–924.

9. Pagano, M., Hoshika, Y., Gennari, F., Manzini, J., Marra, E., Viviano, A., ... & Toncelli, A. (2024). Probing ozone effects on European hornbeam (Carpinus betulus L. and Ostrya carpinifolia Scop.) leaf water content through THz imaging and dynamic stomatal response. *Science of the Total Environment*, *956*, 177358.

10. Manzini, J., Garosi, C., Marra, E., Moura, B. B., Paoletti, E., Hoshika, Y., & Viviano, A. (2025b). Integrating leaf morphological traits can improve the predictive capacity of flux-based ozone metrics for ecophysiological responses in ornamental plant species. *Environmental Pollution*, *384*, 126936.<https://doi.org/10.1016/j.envpol.2025.126936>

11. Hoshika, Y., Cotrozzi, L., Marchica, A., Carrari, E., Lorenzini, G., Nali, C., ... & Pellegrini, E. (2022). Season-long exposure of bilberry plants to realistic and future ozone pollution improves the nutraceutical quality of fruits. *Science of the Total Environment*, *822*, 153577.

12. Manzini, J., Hoshika, Y., Moura, B. B., & Paoletti, E. (2023). Exploring a new O3 index as a proxy for the avoidance/tolerance capacity of forest species to tolerate O3 injury. *Forests*, *14*(5), 901.
